# Moving towards a Robust Definition for a “Healthy” Indoor Microbiome

**DOI:** 10.1128/mSystems.00074-19

**Published:** 2019-05-14

**Authors:** Karen C. Dannemiller

**Affiliations:** a Department of Civil, Environmental & Geodetic Engineering, College of Engineering, Ohio State University, Columbus, Ohio, USA; b Division of Environmental Health Sciences, College of Public Health, Ohio State University, Columbus, Ohio, USA

**Keywords:** building, design, indoor, measurement, microbiome, moisture

## Abstract

Buildings of the future should be designed to support human health, both by promoting the presence of beneficial microbes and by reducing exposure to harmful ones. However, we still do not have a robust definition of what constitutes a “healthy” indoor microbiome. Such a definition would allow us to better understand implications of building design and behavioral decisions of residents, especially for vulnerable populations such as asthmatic children. Relevant assessment methods could then be developed to make microbiome information available to home occupants, environmental health professionals, policy writers, building designers, and building remediation specialists.

## Abstract (precis)

Buildings of the future should be designed to support human health, both by promoting the presence of beneficial microbes and by reducing exposure to harmful ones. However, we still do not have a robust definition of what constitutes a “healthy” indoor microbiome.

## PERSPECTIVE

**FIG 1:**
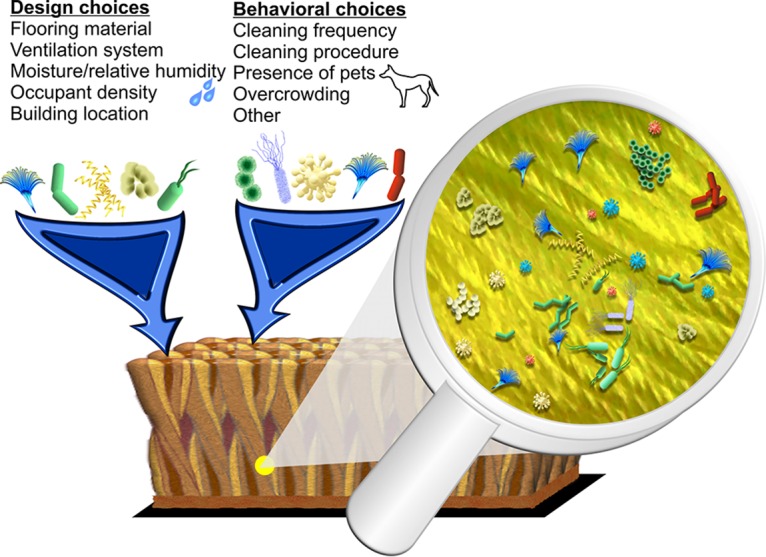
The indoor microbiome represents a diverse group of microorganisms that reside in our homes, as shown in this illustration of carpet. Building design choices (such as flooring material) and behavioral choices (such as cleaning frequency) can influence our daily exposure to different species. (Courtesy of Carl Dannemiller, reproduced with permission.)

The indoor microbiome in each of our homes has important implications for human health. In the future, we can utilize this relationship to promote health through improved building design (Fig. 1). First, there are at least three critical research priorities to address that I have highlighted here. These include defining a healthy indoor microbiome, improving our understanding of how our choices influence the indoor microbiome, and identifying the best assessment method for the indoor microbiome. These will need to be addressed concurrently in an iterative process. This will provide a solid foundation for future development of guidance and standards for buildings to promote health.

## DEFINING A HEALTHY INDOOR MICROBIOME

The World Health Organization defines health as “a state of complete physical, mental and social well-being and not merely the absence of disease or infirmity” (1). Thus, ideally a healthy indoor microbiome is not only one that does not make us sick, but may also promote well-being. We need to consider both positive and negative associations between microorganisms and human health.

Some associations with potentially protective effects have been identified with regard to asthma, allergic sensitization, and wheezing in children. For instance, exposures to microbes associated with animals may be protective against allergy/asthma development (2, 3). Additionally, exposure to high microbial diversity is associated with decreased asthma risk (4, 5). However, microbial richness is a nonspecific measure, and further research is needed to identify what factors are protective. For example, increased richness is sometimes associated with harmful dampness (4), but that is not necessarily inconsistent with the association with health. More work is needed to understand how these exposures vary with regard to health.

Harmful microbial exposures result from excess dampness, moldy odor, and visible mold growth within a building, but the precise causal associations remain unclear (6). The causal factor(s) could be associated with other microbial components related to dampness, including allergen production, mycotoxins, microbial volatile organic compounds, virulence, other factors, or some combinations of these (7, 8). Additionally, pathogen transmission is also a concern in the indoor environment, especially in health care facilities.

Some existing indoor quality standards focus on controlling the microbiome and moisture in buildings, such as the U.S. EPA’s *Moisture Control Guidance for Building Design, Construction and Maintenance* (9) and others (10).

Fully defining a healthy indoor microbiome is likely to be a slow, iterative process. Each microbiome contains thousands of species, which each have diverse microbial functions. Many of these organisms are quite rare, and most may be irrelevant to health outcomes of interest. Additionally, exposures to microbes occur simultaneously with exposures to chemicals, allergens, and pollutants. The effect of these agents may also vary based on characteristics of the population, such as age and diet, and exposure route (ingestion, inhalation, or dermal) and exposure timing may contribute additional complexity to the process. A comprehensive definition of a healthy microbiome must also account for different building types and various building uses.

This complexity presents a complicated statistical challenge. Research is under way to support the development and improvement of tools to evaluate microbial community composition and evaluate longitudinal data to determine changes over time (11).

Ideally, robust delineation of a healthy indoor microbiome will allow future development of standards based on direct microbial measurements.

## IDENTIFYING HOW BUILDING DESIGN, BUILDING CONDITIONS, AND OCCUPANT BEHAVIORAL DECISIONS INFLUENCE THE INDOOR MICROBIOME

Housing characteristics are associated with aspects of microbial communities, including factors such as occupancy, ventilation, location, pets, and moisture (4, 12). Thus, we should have some control over the indoor microbiome by altering building design and behavioral changes, though the extent of this control is still an open question. Many of these factors can also impact health by other means, and a remaining question is if the microbiome is central to these associations. For instance, asthma rates are typically higher in urban compared with less urban areas (5).

Design features and occupant choices could either introduce beneficial microbes or avoid harmful ones. For instance, the introduction of a dog to a home could have a beneficial effect (2), though more work is needed in larger studies to confirm this. An improved understanding of the “healthy microbiome” definition is needed prior to making many of these changes.

Currently, it is more practical to avoid harmful microbial exposures. This could include cleaning surfaces in areas where people are ill to reduce disease transmission through fomites. Eventually, “clean” may evolve from being defined as complete sterilization to being defined as promotion of a beneficial microbiome. We can also avoid moisture and dampness in homes, which are associated with established negative human health effects (6). However, we still need a better understanding of how moisture influences the microbes in homes.

Research continues to build on decades of concerted effort to understand how moisture and dampness in buildings influence microbial communities. More knowledge can be gained in this area through laboratory chamber studies that simulate real-world conditions. Microbiologists have long recognized the need to culture organisms under realistic conditions, as evidenced by the countless variations in types and preparations of culture media. Some of our recent work has demonstrated that microbial growth in carpet dust has the potential to make a substantial contribution to human exposure under elevated relative humidity conditions (13). This work needs to be extended in laboratory chamber studies to more realistic building conditions to evaluate the size of this contribution to human exposure. Additionally, moisture in a building also changes microbial function in carpet dust, and this moisture may also increase exposure to harmful compounds such as allergens (7). These changes in microbial function beg the question, “Can we measure only the presence/absence of species and still see associations with human health, or does the effect of exposure change based on the growth conditions of spores and other fungal fragments?”

Future work will take advantage of new technologies, including metatranscriptomic analysis of fungal communities (7), to yield new and exciting insights into microbial function in our homes and how it may impact our health. In turn, these findings can eventually help promote a healthy indoor microbiome.

## IDENTIFYING ASSESSMENT METHODS FOR THE INDOOR MICROBIOME THAT ARE MOST RELEVANT FOR HUMAN HEALTH

We need to be able to evaluate the indoor microbiome such that we consistently determine its impact in affecting human health. This will serve as a means to ensure that design choices and behavioral decisions lead to intended outcomes. The ideal assessment method would be accessible to environmental health practitioners, of a reasonable cost, time efficient, easy to use, and produce understandable results. However, there are many uncertainties to address before this assessment method can be developed. These uncertainties include when and where to measure, what matrix (surfaces, dust, etc.) to measure, what microbial component to measure, and what to look for in the final results.

We may need to look for exposure in new locations within a home, and we can draw an analogy to studies of lead in housing. One of the major breakthroughs in understanding how lead paint impacts children came from a focus on house dust and wipe sampling (14). Prior to this work, measurements were largely taken from walls and other areas that may not have directly impacted exposure. Finally, sampling the direct source of lead exposure (dust) revealed the associations with blood lead levels in children (15). Lead could then be appropriately measured and regulated. Similarly, we need to carefully select where we measure exposure to microorganisms because microbial communities vary by surface type and human contact levels.

We must also select the most relevant material to measure, which will be highly dependent on the definition of a healthy microbiome and other factors such as building type. Options include airborne particulate matter, volatile organic compounds, settled dust, tape samples from surfaces, areas of active growth, and possibly others.

It is also still unclear which microbial component we need to measure. Some of the strongest negative health associations have been found with visible mold growth and moldy odor (6), which potentially offers clues to possible measurement techniques. High-throughput DNA sequencing of amplicons currently offers the most obvious choice for a microbiome assessment method. The steep drop in cost for DNA sequencing may soon allow this method to be more widespread, but this technique still requires complicated bioinformatics, and processing may not be widely feasible for some time. Metagenomic and metatranscriptomic analyses of communities may also help identify important associations and future measurement targets. These cutting-edge techniques may be too costly and difficult to use on a routine basis by most building scientists and environmental health professionals, but can provide extraordinary insights to identify targets that are ultimately measured by other, more widely accessible methods.

Additionally, we still need to identify which features of the community are the most relevant for human health. Ideally, the results of such a measurement would be easy to understand with suggested actions to remediate any concerns.

## WE NEED TO BE ABLE TO PROVIDE GUIDANCE RELATED TO THE INDOOR MICROBIOME

Ultimately, we need information about the indoor microbiome to be accessible, understandable, and measurable. We can then develop standardized metrics, regulations, and best practices to improve the health and well-being of building occupants. We also need to train scientists of the future in interdisciplinary skills to integrate information from disparate fields for comprehensive recommendations that are both practical and effective (16). This information can then be used to inform public health, policy, and design decisions related to improvements in human health, especially for asthmatics and other vulnerable populations. Such efforts can also be used to reduce health disparities associated with poor-quality housing conditions.

## References

[1] WHO. 1946 Constitution of the World Health Organization. https://www.who.int/governance/eb/who_constitution_en.pdf. Accessed 14 January 2019.

[2] Fujimura KE, Demoor T, Rauch M, Faruqi AA, Jang S, Johnson CC, Boushey HA, Zoratti E, Ownby D, Lukacs NW, Lynch SV 2014 House dust exposure mediates gut microbiome Lactobacillus enrichment and airway immune defense against allergens and virus infection. Proc Natl Acad Sci U S A 111:805–810. doi:10.1073/pnas.1310750111. 24344318 PMC3896155

[3] Stein MM, Hrusch CL, Gozdz J, Igartua C, Pivniouk V, Murray SE, Ledford JG, Marques dos Santos M, Anderson RL, Metwali N, Neilson JW, Maier RM, Gilbert JA, Holbreich M, Thorne PS, Martinez FD, von Mutius E, Vercelli D, Ober C, Sperling AI 2016 Innate immunity and asthma risk in Amish and Hutterite farm children. N Engl J Med 375:411–421. doi:10.1056/NEJMoa1508749. 27518660 PMC5137793

[4] Dannemiller KC, Mendell MJ, Macher JM, Kumagai K, Bradman A, Holland N, Harley K, Eskenazi B, Peccia J 2014 Next-generation DNA sequencing reveals that low fungal diversity in house dust is associated with childhood asthma development. Indoor Air 24:236–247. doi:10.1111/ina.12072. 24883433 PMC4048861

[5] Ege MJ, Mayer M, Schwaiger K, Mattes J, Pershagen G, van Hage M, Scheynius A, Bauer J, von Mutius E 2012 Environmental bacteria and childhood asthma. Allergy 67:1565–1571. doi:10.1111/all.12028. 22994424

[6] Mendell MJ, Mirer AG, Cheung K, Tong M, Douwes J 2011 Respiratory and allergic health effects of dampness, mold, and dampness-related agents: a review of the epidemiologic evidence. Environ Health Perspect 119:748–756. doi:10.1289/ehp.1002410. 21269928 PMC3114807

[7] Hegarty B, Dannemiller K, Peccia J 2018 Gene expression of indoor fungal communities under damp building conditions: implications for human health. Indoor Air 28:548–558. doi:10.1111/ina.12459. 29500849

[8] Korpi A, Pasanen AL, Pasanen P, Kalliokoski P 1997 Microbial growth and metabolism in house dust. Int Biodeterior Biodegrad 40:19–27. doi:10.1016/S0964-8305(97)00032-2.

[9] US Environmental Protection Agency. 2013 Moisture control guidance for building design, construction and maintenance. US Environmental Protection Agency, Washington, DC.

[10] Bope A, Weir MH, Pruden A, Morowitz M, Mitchell J, Dannemiller KC 2018 Translating research to policy at the NCSE 2017 symposium Microbiology of the Built Environment: Implications for Health and Design. Microbiome 6:160. doi:10.1186/s40168-018-0552-y. 30219094 PMC6138931

[11] Xia Y, Sun J 2017 Hypothesis testing and statistical analysis of microbiome. Genes Dis 4:138–148. doi:10.1016/j.gendis.2017.06.001. 30197908 PMC6128532

[12] Dannemiller KC, Gent JF, Leaderer BP, Peccia J 2017 Influence of housing characteristics on bacterial and fungal communities in homes of asthmatic children. Indoor Air 26:179–192. doi:10.1111/ina.12205. PMC4591094 25833176

[13] Dannemiller KC, Weschler CJ, Peccia J 2017 Fungal and bacterial growth in floor dust at elevated relative humidity levels. Indoor Air 27:354–363. doi:10.1111/ina.12313. 27272645

[14] Lanphear BP, Emond M, Jacobs DE, Weitzman M, Tanner M, Winter NL, Yakir B, Eberly S 1995 A side-by-side comparison of dust collection methods for sampling lead-contaminated house dust. Environ Res 68:114–123. doi:10.1006/enrs.1995.1015. 7601072

[15] Lanphear BP, Matte TD, Rogers J, Clickner RP, Dietz B, Bornschein RL, Succop P, Mahaffey KR, Dixon S, Galke W, Rabinowitz M, Farfel M, Rohde C, Schwartz J, Ashley P, Jacobs DE 1998 The contribution of lead-contaminated house dust and residential soil to children's blood lead levels. Environ Res 79:51–68. doi:10.1006/enrs.1998.3859. 9756680

[16] Dannemiller KC 2019 Engineering design for environmental health: a new course preparing students to address interdisciplinary challenges. Environ Eng Sci 36:257–261. doi:10.1089/ees.2018.0284.

